# 3-[(1-Benzyl-1*H*-1,2,3-triazol-4-yl)methyl]-1,5-dimethyl-1,5-benzodiazepine-2,4-dione

**DOI:** 10.1107/S1600536810005313

**Published:** 2010-02-13

**Authors:** R. Dardouri, Y. Kandri Rodi, Natalie Saffon, El Mokhtar Essassi, Seik Weng Ng

**Affiliations:** a Laboratoire de Chimie Organique Appliquée, Faculté des Sciences et Techniques, Université Sidi Mohamed Ben Abdallah, Fés, Morocco; b Service Commun Rayons-X FR2599, Université Paul Sabatier Bâtiment 2R1, 118 Route de Narbonne, Toulouse, France; c Laboratoire de Chimie Organique Hétérocyclique, Pôle de Compétences Pharmacochimie, Université Mohammed V-Agdal, BP 1014 Avenue Ibn Batout, Rabat, Morocco; d Department of Chemistry, University of Malaya, 50603 Kuala Lumpur, Malaysia

## Abstract

The title compound, C_21_H_21_N_5_O_2_, is a 1,4-dimethyl-1,2,3-triazole having dimethyl­benzodiazepindione and phenyl substituents on each methyl group; the substituents are positioned on opposite sides of the five-membered ring. The seven-membered fused-ring of the larger substituent adopts a boat-shaped conformation (with the methine C atom as the prow).

## Related literature

For the crystal structure of 1,5-dimethyl-1,5-benzodiazepin-2,4-dione, see: Mondieig *et al.* (2005 ▶).
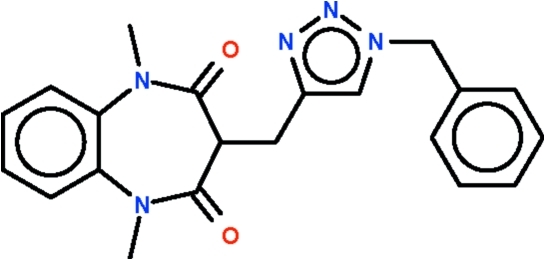


## Experimental

### 

#### Crystal data

C_21_H_21_N_5_O_2_
*M* *_r_* = 375.43
Triclinic, 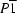

*a* = 8.3380 (3) Å
*b* = 9.1033 (3) Å
*c* = 13.4796 (4) Å
α = 95.385 (2)°
β = 107.840 (2)°
γ = 101.768 (2)°
*V* = 939.97 (5) Å^3^
*Z* = 2
Mo *K*α radiation
μ = 0.09 mm^−1^
*T* = 293 K
0.28 × 0.18 × 0.08 mm

#### Data collection

Bruker APEXII diffractometer
7973 measured reflections
3279 independent reflections
2132 reflections with *I* > 2σ(*I*)
*R*_int_ = 0.037

#### Refinement

*R*[*F* ^2^ > 2σ(*F* ^2^)] = 0.042
*wR*(*F* ^2^) = 0.141
*S* = 1.05
3279 reflections
255 parameters
H-atom parameters constrained
Δρ_max_ = 0.22 e Å^−3^
Δρ_min_ = −0.22 e Å^−3^

### 

Data collection: *APEX2* (Bruker, 2005 ▶); cell refinement: *SAINT* (Bruker, 2005 ▶); data reduction: *SAINT*; program(s) used to solve structure: *SHELXS97* (Sheldrick, 2008 ▶); program(s) used to refine structure: *SHELXL97* (Sheldrick, 2008 ▶); molecular graphics: *X-SEED* (Barbour, 2001 ▶); software used to prepare material for publication: *publCIF* (Westrip, 2010 ▶).

## Supplementary Material

Crystal structure: contains datablocks global, I. DOI: 10.1107/S1600536810005313/bt5192sup1.cif

Structure factors: contains datablocks I. DOI: 10.1107/S1600536810005313/bt5192Isup2.hkl

Additional supplementary materials:  crystallographic information; 3D view; checkCIF report

## Figures and Tables

**Fig. 1.**
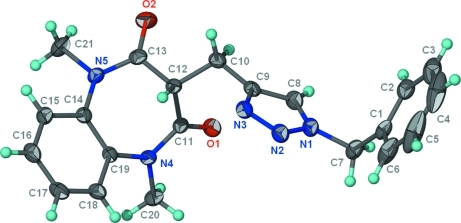
Thermal ellipsoid plot (Barbour, 2001) of C21H21N5O2 at the 50% probability level; hydrogen atoms are drawn as arbitrary radius.

## supplementary crystallographic information

### Experimental

To a solution of 1,5-dimethyl-3-propargyl-1,5-benzodiazepine-2,4-dione (1 mmol) in *t*-butyl alcohol/water (1/2, 8 ml) was added copper sulfate pentahydrate (1 mmol), sodium ascorbate (2 mmol) and benzyl azide (5 mmol). The mixture was stirred for 8 h. The solution was then diluted with water (20 ml) and the organic compound extracted with ethyl acetate (2 × 20 ml). The extracts were washed with brine and dried over sodium sulfate. The compound was recrystallized from ether to give colorless crystals.

### Refinement

Carbon-bound H atoms were placed in calculated positions (C—H 0.93–0.98 Å) and were included in the refinement in the riding model approximation, with *U*(H) set to 1.2–1.5*U*(C).

### Crystal data

**Table:**

C_21_H_21_N_5_O_2_	*Z* = 2
*M**_r_* = 375.43	*F*(000) = 396
Triclinic, *P*1	*D*_x_ = 1.326 Mg m^−^^3^
Hall symbol: -P 1	Mo *K*α radiation, λ = 0.71073 Å
*a* = 8.3380 (3) Å	Cell parameters from 1894 reflections
*b* = 9.1033 (3) Å	θ = 2.3–26.8°
*c* = 13.4796 (4) Å	µ = 0.09 mm^−^^1^
α = 95.385 (2)°	*T* = 293 K
β = 107.840 (2)°	Block, colourless
γ = 101.768 (2)°	0.28 × 0.18 × 0.08 mm
*V* = 939.97 (5) Å^3^	

### Data collection

**Table:**

Bruker APEXII diffractometer	2132 reflections with *I* > 2σ(*I*)
Radiation source: fine-focus sealed tube	*R*_int_ = 0.037
graphite	θ_max_ = 25.0°, θ_min_ = 1.6°
φ and ω scans	*h* = −8→9
7973 measured reflections	*k* = −10→10
3279 independent reflections	*l* = −16→15

### Refinement

**Table:**

Refinement on *F*^2^	Primary atom site location: structure-invariant direct methods
Least-squares matrix: full	Secondary atom site location: difference Fourier map
*R*[*F*^2^ > 2σ(*F*^2^)] = 0.042	Hydrogen site location: inferred from neighbouring sites
*wR*(*F*^2^) = 0.141	H-atom parameters constrained
*S* = 1.05	*w* = 1/[σ^2^(*F*_o_^2^) + (0.076*P*)^2^] where *P* = (*F*_o_^2^ + 2*F*_c_^2^)/3
3279 reflections	(Δ/σ)_max_ = 0.001
255 parameters	Δρ_max_ = 0.22 e Å^−^^3^
0 restraints	Δρ_min_ = −0.22 e Å^−^^3^

### Fractional atomic coordinates and isotropic or equivalent isotropic displacement parameters (Å^2^)

**Table:**

	*x*	*y*	*z*	*U*_iso_*/*U*_eq_	
O1	0.50707 (19)	0.14271 (17)	0.38773 (13)	0.0383 (4)	
O2	0.8254 (2)	0.2153 (2)	0.23619 (15)	0.0501 (5)	
N1	0.8448 (2)	0.2106 (2)	0.71289 (16)	0.0366 (5)	
N2	0.9062 (2)	0.3607 (2)	0.71872 (16)	0.0376 (5)	
N3	0.9231 (2)	0.3802 (2)	0.62613 (16)	0.0359 (5)	
N4	0.4763 (2)	0.37357 (19)	0.34845 (14)	0.0283 (4)	
N5	0.6925 (2)	0.4101 (2)	0.21476 (15)	0.0310 (5)	
C1	0.9776 (3)	0.1642 (3)	0.89419 (19)	0.0359 (6)	
C2	1.0765 (3)	0.0598 (3)	0.8911 (2)	0.0409 (6)	
H2	1.0396	−0.0190	0.8339	0.049*	
C3	1.2289 (4)	0.0720 (4)	0.9720 (3)	0.0650 (9)	
H3	1.2943	0.0013	0.9694	0.078*	
C4	1.2843 (5)	0.1865 (6)	1.0557 (3)	0.0935 (15)	
H4	1.3868	0.1928	1.1106	0.112*	
C5	1.1909 (6)	0.2932 (5)	1.0604 (3)	0.0947 (16)	
H5	1.2312	0.3731	1.1171	0.114*	
C6	1.0353 (5)	0.2807 (3)	0.9796 (2)	0.0610 (9)	
H6	0.9699	0.3512	0.9831	0.073*	
C7	0.8123 (3)	0.1525 (3)	0.8049 (2)	0.0442 (7)	
H7A	0.7476	0.0468	0.7836	0.053*	
H7B	0.7415	0.2096	0.8294	0.053*	
C8	0.8216 (3)	0.1340 (3)	0.6177 (2)	0.0364 (6)	
H8	0.7799	0.0296	0.5944	0.044*	
C9	0.8726 (3)	0.2424 (3)	0.56202 (19)	0.0321 (6)	
C10	0.8729 (3)	0.2271 (3)	0.45103 (19)	0.0346 (6)	
H10A	0.8279	0.1209	0.4181	0.042*	
H10B	0.9915	0.2590	0.4515	0.042*	
C11	0.5716 (3)	0.2698 (2)	0.37552 (18)	0.0282 (5)	
C12	0.7630 (3)	0.3225 (2)	0.38576 (18)	0.0284 (5)	
H12	0.8070	0.4292	0.4202	0.034*	
C13	0.7673 (3)	0.3110 (3)	0.27368 (19)	0.0324 (6)	
C14	0.6468 (3)	0.5362 (2)	0.26045 (17)	0.0286 (5)	
C15	0.6992 (3)	0.6803 (3)	0.2368 (2)	0.0357 (6)	
H15	0.7607	0.6919	0.1898	0.043*	
C16	0.6620 (3)	0.8055 (3)	0.2814 (2)	0.0403 (6)	
H16	0.6977	0.9004	0.2641	0.048*	
C17	0.5715 (3)	0.7908 (3)	0.3521 (2)	0.0385 (6)	
H17	0.5500	0.8760	0.3844	0.046*	
C18	0.5137 (3)	0.6485 (2)	0.37394 (19)	0.0324 (5)	
H18	0.4513	0.6384	0.4205	0.039*	
C19	0.5468 (3)	0.5196 (2)	0.32765 (17)	0.0268 (5)	
C20	0.2922 (3)	0.3329 (3)	0.3420 (2)	0.0364 (6)	
H20A	0.2357	0.2351	0.2985	0.055*	
H20B	0.2356	0.4080	0.3118	0.055*	
H20C	0.2857	0.3293	0.4117	0.055*	
C21	0.7053 (3)	0.4078 (3)	0.1087 (2)	0.0440 (7)	
H21A	0.8197	0.4626	0.1138	0.066*	
H21B	0.6207	0.4549	0.0672	0.066*	
H21C	0.6841	0.3044	0.0756	0.066*	

### Atomic displacement parameters (Å^2^)

**Table:**

	*U*^11^	*U*^22^	*U*^33^	*U*^12^	*U*^13^	*U*^23^
O1	0.0331 (9)	0.0258 (9)	0.0594 (12)	0.0034 (7)	0.0207 (8)	0.0141 (8)
O2	0.0635 (12)	0.0451 (11)	0.0596 (12)	0.0259 (9)	0.0373 (10)	0.0104 (9)
N1	0.0281 (10)	0.0379 (12)	0.0463 (13)	0.0062 (9)	0.0139 (9)	0.0187 (10)
N2	0.0316 (11)	0.0349 (12)	0.0476 (13)	0.0078 (9)	0.0133 (10)	0.0139 (9)
N3	0.0278 (10)	0.0370 (12)	0.0461 (13)	0.0085 (9)	0.0138 (9)	0.0160 (10)
N4	0.0219 (9)	0.0264 (10)	0.0388 (12)	0.0037 (8)	0.0138 (8)	0.0080 (8)
N5	0.0315 (10)	0.0314 (11)	0.0333 (11)	0.0061 (8)	0.0163 (9)	0.0067 (9)
C1	0.0399 (13)	0.0350 (14)	0.0344 (14)	−0.0005 (11)	0.0197 (11)	0.0095 (11)
C2	0.0387 (14)	0.0420 (15)	0.0435 (16)	0.0041 (11)	0.0176 (12)	0.0138 (12)
C3	0.0380 (16)	0.089 (2)	0.070 (2)	0.0062 (16)	0.0172 (16)	0.048 (2)
C4	0.061 (2)	0.113 (3)	0.060 (3)	−0.043 (2)	−0.0130 (18)	0.048 (3)
C5	0.138 (4)	0.069 (3)	0.0316 (19)	−0.049 (3)	0.014 (2)	0.0020 (17)
C6	0.099 (2)	0.0374 (16)	0.0501 (19)	−0.0039 (15)	0.0430 (19)	0.0055 (13)
C7	0.0399 (14)	0.0494 (16)	0.0526 (17)	0.0084 (12)	0.0261 (13)	0.0233 (13)
C8	0.0334 (13)	0.0277 (13)	0.0478 (16)	0.0042 (10)	0.0139 (12)	0.0108 (11)
C9	0.0200 (11)	0.0302 (13)	0.0494 (15)	0.0079 (9)	0.0128 (10)	0.0147 (11)
C10	0.0288 (12)	0.0313 (13)	0.0501 (16)	0.0102 (10)	0.0185 (11)	0.0132 (11)
C11	0.0291 (12)	0.0243 (12)	0.0338 (13)	0.0043 (9)	0.0153 (10)	0.0067 (9)
C12	0.0272 (11)	0.0234 (11)	0.0385 (14)	0.0054 (9)	0.0162 (10)	0.0074 (10)
C13	0.0292 (12)	0.0279 (13)	0.0432 (15)	0.0034 (10)	0.0190 (11)	0.0055 (11)
C14	0.0223 (11)	0.0300 (12)	0.0331 (13)	0.0059 (9)	0.0084 (10)	0.0081 (10)
C15	0.0267 (12)	0.0379 (14)	0.0472 (15)	0.0062 (10)	0.0170 (11)	0.0185 (12)
C16	0.0310 (13)	0.0294 (13)	0.0630 (18)	0.0065 (10)	0.0165 (12)	0.0194 (12)
C17	0.0321 (13)	0.0273 (13)	0.0577 (17)	0.0101 (10)	0.0153 (12)	0.0076 (11)
C18	0.0254 (11)	0.0316 (13)	0.0411 (14)	0.0088 (10)	0.0109 (10)	0.0068 (10)
C19	0.0229 (11)	0.0244 (12)	0.0322 (13)	0.0053 (9)	0.0078 (9)	0.0070 (9)
C20	0.0234 (12)	0.0421 (14)	0.0460 (15)	0.0032 (10)	0.0164 (11)	0.0116 (11)
C21	0.0493 (15)	0.0498 (16)	0.0386 (15)	0.0088 (12)	0.0242 (13)	0.0103 (12)

### Geometric parameters (Å, °)

**Table:**

O1—C11	1.221 (2)	C7—H7B	0.9700
O2—C13	1.221 (3)	C8—C9	1.372 (3)
N1—C8	1.340 (3)	C8—H8	0.9300
N1—N2	1.344 (3)	C9—C10	1.490 (3)
N1—C7	1.470 (3)	C10—C12	1.531 (3)
N2—N3	1.322 (3)	C10—H10A	0.9700
N3—C9	1.362 (3)	C10—H10B	0.9700
N4—C11	1.361 (3)	C11—C12	1.528 (3)
N4—C19	1.429 (3)	C12—C13	1.517 (3)
N4—C20	1.477 (3)	C12—H12	0.9800
N5—C13	1.376 (3)	C14—C15	1.394 (3)
N5—C14	1.425 (3)	C14—C19	1.406 (3)
N5—C21	1.464 (3)	C15—C16	1.374 (3)
C1—C6	1.380 (4)	C15—H15	0.9300
C1—C2	1.385 (3)	C16—C17	1.385 (3)
C1—C7	1.503 (4)	C16—H16	0.9300
C2—C3	1.374 (4)	C17—C18	1.379 (3)
C2—H2	0.9300	C17—H17	0.9300
C3—C4	1.355 (6)	C18—C19	1.394 (3)
C3—H3	0.9300	C18—H18	0.9300
C4—C5	1.371 (6)	C20—H20A	0.9600
C4—H4	0.9300	C20—H20B	0.9600
C5—C6	1.390 (5)	C20—H20C	0.9600
C5—H5	0.9300	C21—H21A	0.9600
C6—H6	0.9300	C21—H21B	0.9600
C7—H7A	0.9700	C21—H21C	0.9600
			
C8—N1—N2	111.21 (19)	C12—C10—H10B	109.2
C8—N1—C7	129.2 (2)	H10A—C10—H10B	107.9
N2—N1—C7	119.6 (2)	O1—C11—N4	122.12 (19)
N3—N2—N1	106.65 (19)	O1—C11—C12	122.85 (19)
N2—N3—C9	109.22 (18)	N4—C11—C12	115.00 (18)
C11—N4—C19	123.05 (17)	C13—C12—C11	105.92 (18)
C11—N4—C20	117.89 (17)	C13—C12—C10	112.28 (18)
C19—N4—C20	119.06 (17)	C11—C12—C10	111.64 (17)
C13—N5—C14	122.66 (19)	C13—C12—H12	109.0
C13—N5—C21	116.48 (19)	C11—C12—H12	109.0
C14—N5—C21	118.77 (18)	C10—C12—H12	109.0
C6—C1—C2	118.8 (3)	O2—C13—N5	121.9 (2)
C6—C1—C7	120.7 (3)	O2—C13—C12	122.3 (2)
C2—C1—C7	120.4 (2)	N5—C13—C12	115.6 (2)
C3—C2—C1	120.4 (3)	C15—C14—C19	118.5 (2)
C3—C2—H2	119.8	C15—C14—N5	119.6 (2)
C1—C2—H2	119.8	C19—C14—N5	121.94 (19)
C4—C3—C2	120.4 (3)	C16—C15—C14	121.4 (2)
C4—C3—H3	119.8	C16—C15—H15	119.3
C2—C3—H3	119.8	C14—C15—H15	119.3
C3—C4—C5	120.7 (3)	C15—C16—C17	120.2 (2)
C3—C4—H4	119.7	C15—C16—H16	119.9
C5—C4—H4	119.7	C17—C16—H16	119.9
C4—C5—C6	119.4 (3)	C18—C17—C16	119.3 (2)
C4—C5—H5	120.3	C18—C17—H17	120.4
C6—C5—H5	120.3	C16—C17—H17	120.4
C1—C6—C5	120.3 (3)	C17—C18—C19	121.4 (2)
C1—C6—H6	119.9	C17—C18—H18	119.3
C5—C6—H6	119.9	C19—C18—H18	119.3
N1—C7—C1	112.38 (19)	C18—C19—C14	119.15 (19)
N1—C7—H7A	109.1	C18—C19—N4	119.24 (19)
C1—C7—H7A	109.1	C14—C19—N4	121.60 (19)
N1—C7—H7B	109.1	N4—C20—H20A	109.5
C1—C7—H7B	109.1	N4—C20—H20B	109.5
H7A—C7—H7B	107.9	H20A—C20—H20B	109.5
N1—C8—C9	105.3 (2)	N4—C20—H20C	109.5
N1—C8—H8	127.4	H20A—C20—H20C	109.5
C9—C8—H8	127.4	H20B—C20—H20C	109.5
N3—C9—C8	107.7 (2)	N5—C21—H21A	109.5
N3—C9—C10	122.0 (2)	N5—C21—H21B	109.5
C8—C9—C10	130.3 (2)	H21A—C21—H21B	109.5
C9—C10—C12	112.07 (18)	N5—C21—H21C	109.5
C9—C10—H10A	109.2	H21A—C21—H21C	109.5
C12—C10—H10A	109.2	H21B—C21—H21C	109.5
C9—C10—H10B	109.2		
			
C8—N1—N2—N3	−0.3 (2)	N4—C11—C12—C10	160.55 (19)
C7—N1—N2—N3	179.89 (18)	C9—C10—C12—C13	178.25 (18)
N1—N2—N3—C9	0.0 (2)	C9—C10—C12—C11	−63.0 (2)
C6—C1—C2—C3	0.4 (3)	C14—N5—C13—O2	−170.7 (2)
C7—C1—C2—C3	179.0 (2)	C21—N5—C13—O2	−7.4 (3)
C1—C2—C3—C4	−0.2 (4)	C14—N5—C13—C12	12.8 (3)
C2—C3—C4—C5	−0.9 (5)	C21—N5—C13—C12	176.14 (18)
C3—C4—C5—C6	1.8 (5)	C11—C12—C13—O2	−109.6 (2)
C2—C1—C6—C5	0.5 (4)	C10—C12—C13—O2	12.5 (3)
C7—C1—C6—C5	−178.1 (2)	C11—C12—C13—N5	66.9 (2)
C4—C5—C6—C1	−1.6 (5)	C10—C12—C13—N5	−171.04 (18)
C8—N1—C7—C1	110.1 (3)	C13—N5—C14—C15	130.3 (2)
N2—N1—C7—C1	−70.1 (3)	C21—N5—C14—C15	−32.6 (3)
C6—C1—C7—N1	100.6 (3)	C13—N5—C14—C19	−50.9 (3)
C2—C1—C7—N1	−78.0 (3)	C21—N5—C14—C19	146.1 (2)
N2—N1—C8—C9	0.5 (2)	C19—C14—C15—C16	3.0 (3)
C7—N1—C8—C9	−179.7 (2)	N5—C14—C15—C16	−178.2 (2)
N2—N3—C9—C8	0.3 (2)	C14—C15—C16—C17	0.4 (4)
N2—N3—C9—C10	178.56 (19)	C15—C16—C17—C18	−2.4 (4)
N1—C8—C9—N3	−0.5 (2)	C16—C17—C18—C19	1.0 (3)
N1—C8—C9—C10	−178.5 (2)	C17—C18—C19—C14	2.4 (3)
N3—C9—C10—C12	−56.4 (3)	C17—C18—C19—N4	−176.7 (2)
C8—C9—C10—C12	121.4 (2)	C15—C14—C19—C18	−4.3 (3)
C19—N4—C11—O1	−176.2 (2)	N5—C14—C19—C18	176.90 (19)
C20—N4—C11—O1	4.7 (3)	C15—C14—C19—N4	174.74 (18)
C19—N4—C11—C12	1.9 (3)	N5—C14—C19—N4	−4.0 (3)
C20—N4—C11—C12	−177.20 (19)	C11—N4—C19—C18	−132.9 (2)
O1—C11—C12—C13	101.1 (2)	C20—N4—C19—C18	46.1 (3)
N4—C11—C12—C13	−76.9 (2)	C11—N4—C19—C14	48.0 (3)
O1—C11—C12—C10	−21.4 (3)	C20—N4—C19—C14	−132.9 (2)
